# Improving prediction models with new markers: a comparison of updating strategies

**DOI:** 10.1186/s12874-016-0231-2

**Published:** 2016-09-27

**Authors:** D. Nieboer, Y. Vergouwe, Danna P. Ankerst, Monique J. Roobol, Ewout W. Steyerberg

**Affiliations:** 1 Department of Public Health, Erasmus MC-University Medical Center Rotterdam, P.O. box 2040, 3000 Rotterdam, CA The Netherlands; 2 Department of Mathematics, Technical University Munich, Munich, Germany; 3 University of Texas Health Science Center at San Antonio, San Antonio, TX USA; 4 Department of Urology, Erasmus MC-University Medical Center Rotterdam, Rotterdam, The Netherlands

**Keywords:** Prediction model, Prostate cancer, Model updating, Logistic regression

## Abstract

**Background:**

New markers hold the promise of improving risk prediction for individual patients. We aimed to compare the performance of different strategies to extend a previously developed prediction model with a new marker.

**Methods:**

Our motivating example was the extension of a risk calculator for prostate cancer with a new marker that was available in a relatively small dataset. Performance of the strategies was also investigated in simulations. Development, marker and test sets with different sample sizes originating from the same underlying population were generated. A prediction model was fitted using logistic regression in the development set, extended using the marker set and validated in the test set. Extension strategies considered were re-estimating individual regression coefficients, updating of predictions using conditional likelihood ratios (LR) and imputation of marker values in the development set and subsequently fitting a model in the combined development and marker sets. Sample sizes considered for the development and marker set were 500 and 100, 500 and 500, and 100 and 500 patients. Discriminative ability of the extended models was quantified using the concordance statistic (*c*-statistic) and calibration was quantified using the calibration slope.

**Results:**

All strategies led to extended models with increased discrimination (c-statistic increase from 0.75 to 0.80 in test sets). Strategies estimating a large number of parameters (re-estimation of all coefficients and updating using conditional LR) led to overfitting (calibration slope below 1). Parsimonious methods, limiting the number of coefficients to be re-estimated, or applying shrinkage after model revision, limited the amount of overfitting. Combining the development and marker set using imputation of missing marker values approach led to consistently good performing models in all scenarios. Similar results were observed in the motivating example.

**Conclusion:**

When the sample with the new marker information is small, parsimonious methods are required to prevent overfitting of a new prediction model. Combining all data with imputation of missing marker values is an attractive option, even if a relatively large marker data set is available.

**Electronic supplementary material:**

The online version of this article (doi:10.1186/s12874-016-0231-2) contains supplementary material, which is available to authorized users.

## Background

Markers for disease risk, such as genetic characteristics, imaging, and biomarkers, may be useful to improve clinical prediction models. Incorporating markers in multivariable prediction models should lead to better individualized risk estimates, such that more personalized medicine is achieved [1–3]. Data sets with new marker data are however often relatively small [4]. This poses a challenge since overfitting may easily occur in developing prediction models with limited sample size [5]. A new model with marker data incorporated may then perform worse than a model without, if the latter was based on a substantially larger data set.

Developing a prediction model with limited sample size may lead to too optimistic estimates of predictor effects [6, 7]. Optimistic estimates of predictor effects lead to poor calibration of a prediction model when applied in new patients. Applying shrinkage techniques may limit this problem. In the same spirit as shrinkage, one may consider updating existing prediction models using parsimonious methods rather than refitting all model parameters [8]. Parsimonious updating methods consider fewer parameters that need to be estimated, which is especially relevant in small samples.

Recently, a method was proposed that uses conditional likelihood ratios (CLRs) for extension of an existing prediction model. The CLRs are calculated for the marker values conditional on the predictors in the existing prediction model [9]. The predictions of the existing prediction model are then updated by combining them with the CLRs using Bayes rule.

If individual patient data from the development dataset on which the existing prediction was developed are available, this set can be combined with the new dataset containing information on the new marker. The advantage of this approach is that the extended model may exploit all available data on the predictor effects, although the marker values in the development set are systematically missing. After (multiple) imputation of the missing marker values, an extended prediction model can be developed based on the combined development and marker set.

In this study, we aimed to investigate the performance of different strategies of extending an existing prediction model with a new marker. We specifically focused on the impact of small sample size of the marker set in simulation studies and risks of overfitting. We first introduce a motivating example of men at risk for prostate cancer, followed by a description of various strategies to extend an existing model and results from a simulation study. We conclude with a discussion of our findings and recommendations for the situation that the development data set is or is not available at the time of model updating.

## Methods

### Motivating example

**Fig. 1:**
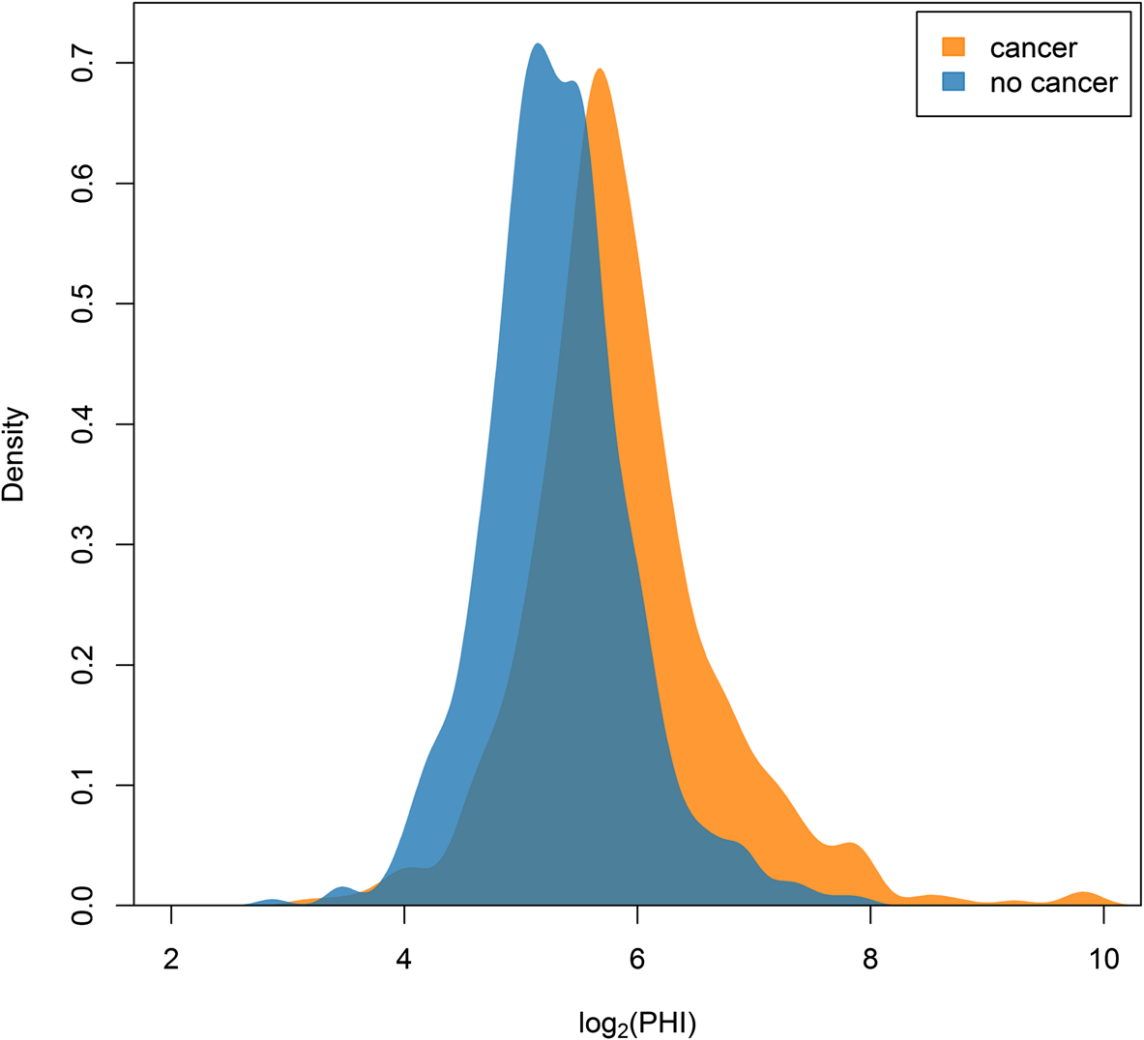
Density estimate of PHI levels for cases and controls in the marker set

The European Randomized Study of Prostate Cancer (ERSPC) is a large randomized study that provided the basis for a number of clinical prediction models, presented as risk calculators (RCs) [10–12]. One such risk calculator (“ERSPC RC3”) estimates the probability of a positive sextant biopsy in previously unscreened men based on three clinical characteristics: prostate-specific antigen (PSA, a continuous variable), prostate volume (a continuous variable), and the result of a digital rectal exam (DRE, a binary variable) [12]. The ERSPC RC3 was developed on a cohort of 3,624 previously un-biopsied men (Table 1). Recently the Prostate Health Index (PHI) has been proposed as a promising marker, which should help to better discriminate between patients with and without prostate cancer (Fig. 1) [13, 14]. We aimed to extend the ERSPC RC3 with the new marker PHI. Data were available from five European sites that collected PHI in addition to the same variables used by ERSPC RC3 (*n* = 1,243).

**Table 1:** Characteristics of patients used at the development of ERSPC RC3 (ERSPC section Rotterdam) and characteristics of patients collected at 5 different sites in Europe with information on the additional marker PHI

Variable	Measure or category	ERSPC Rotterdam	Paris	Rennes	Munster	Hamburg	Milan
		*n* = 3,616	*n* = 108	*n* = 188	*n* = 319	*n* = 182	*n* = 446
PSA (ng/ml)	Median (25–75 percentile)	3.1 (2.8–3.6)	4.4 (3.5–5.6)	4.6 (3.5–5.8)	5.1 (4.1–6.4)	6.5 (4.4–9.6)	5.4 (4.2–7.0)
Prostate Volume	25 cc	739 (20 %)	22 (20 %)	48 (26 %)	78 (24 %)	83 (46 %)	271 (61 %)
	40 cc	1728 (49 %)	59 (55 %)	60 (32 %)	96 (30 %)	23 (13 %)	140 (31 %)
	60 cc	1149 (32 %)	27 (25 %)	80 (43 %)	145 (45 %)	76 (42 %)	35 (8 %)
Abnormal DRE	Yes	1279 (35 %)	40 (37 %)	120 (64 %)	49 (15 %)	52 (29 %)	76 (17 %)
PHI	Median (25–75 percentile)	–	45 (34–61)	68 (42–121)	45 (32–59)	40 (30–61)	47 (37–57)
Cancer	Yes	885 (24 %)	62 (57 %)	107 (57 %)	184 (58 %)	100 (55 %)	187 (42 %)

All datasets containing information on PHI showed higher proportions of patients with cancer (42–58 %) compared to the development set (24 %) (Table 1). The marker set from Hamburg showed the highest PSA levels (median 6.5 ng/l), which were far above the PSA levels in the ERSPC development set (median 3.1 ng/l).

We extended the ERSPC RC3 using data from one site to simulate the situation that only a small sample of patients with PHI is available. The extended ERSPC RC3 was subsequently validated in the data from the four sites not used at model extension.

### Strategies to extend a prediction model

We considered several strategies to extend an existing prediction model developed using logistic regression with a new marker (Table 2). The first method was not to allow for any updating. This was considered the reference on which the extended models needed to improve upon. The linear predictor lp_0_ of the existing, previously developed, prediction model is given by:

**Table 2:** Characteristics of the update methods and number of parameters estimated in the case study of prediction of prostate cancer at biopsy

Method	Data required	Nr. Parameters estimated	Nr. Parameters estimated in case study
Original Model	No data	0	0
Model Revision with extension	Marker set	*p* + *m* + 1	5
Model Revision with shrinkage	Marker set	*p* + *m* + 1	5
Recalibration and extension	Marker set	*m* + 2	3
CLR	Marker set	2 m (*p* + *1*) + m (m + 1)	10
CLR simple	Marker set	*m* (*p* + 2) + m (m + 1)/2	6
Imputation	Development and marker	*p* + *m* + *1*	5

*p*: number of predictors in original model, *m*: number of markers

$$ {\mathrm{lp}}_0=\alpha +{\displaystyle {\sum}_{i=1}^p{\beta}_i{x}_i}, $$

where α is the model intercept, β_*i*_ are the regression coefficients as available for the existing model, and *x*_*i*_ the predictors, with values from the new data set. Various strategies for extending an existing prediction model with a new marker were considered which fell into 3 classes: re-estimation of all regression coefficients, including the coefficient for the new marker; Bayesian updating of predictions using conditional likelihood ratios; and imputation (Table 2). Re-estimation of the regression coefficients and Bayesian updating of predictions using conditional likelihood ratios only require the marker set, while the imputation approach also requires the availability of the development set of the original prediction model.

### Re-estimation of regression coefficients

A straightforward way of extending the existing prediction model would be to fit a logistic regression model to the new marker data set only, containing the same variables as required for the existing model in addition to the new marker as predictors. We label this strategy “model revision with extension” [8, 15]. The linear predictor lp_1_ of this method becomes

$$ {\mathrm{lp}}_1=\widehat{\alpha}+{\displaystyle {\sum}_{i=1}^p{\widehat{\beta}}_i}\;{x}_i+{\widehat{\beta}}_{p+1}\;m, $$

where *m* is the novel marker, and the hats above parameters denote the associated estimated regression coefficients. Model revision with extension requires the estimation of *p* + 2 parameters using data from the marker set. In relatively small marker datasets, this strategy may suffer from a tendency to overfit [6, 7]. To mitigate these problems, we additionally consider shrinkage of the refitted coefficients towards the recalibrated regression coefficients (“model extension with shrinkage”) [8]. The regression coefficient of the new marker is shrunken towards zero. The recalibrated regression coefficients are obtained by first fitting a logistic regression model with only a single covariate, i.e. the linear predictor of the original prediction model, lp_0_, to the new marker dataset, yielding ${\mathrm{lp}}_2=\widehat{\alpha}+{\widehat{\beta}}_{\mathrm{overall}}{\mathrm{lp}}_0$. The linear predictor of “model revision with shrinkage” is given by

$$ {\mathrm{lp}}_3=\widehat{c}{\mathrm{lp}}_1+\left(1-\widehat{c}\right){\mathrm{lp}}_2, $$

where *ĉ* is the heuristic shrinkage factor estimated by

$$ \widehat{c}=\frac{ \max \left({\chi}_{\mathrm{revision}-\mathrm{recalibrated}}^2-\mathrm{d}\mathrm{f},0\right)}{\chi_{\mathrm{revision}-\mathrm{recalibrated}}^2}, $$

*χ*_revision − recalibrated_^2^ is the difference in-2 log-likelihood between the extended and recalibrated model, and df is the difference in degrees of freedom of the extended and recalibrated model (*p* − 1 in our case).

Over fitting may also be limited by reducing the number of estimated parameters. Therefore, we considered a third strategy by including the new marker in the recalibrated prediction model (“recalibration with extension”). Regression coefficients were estimated by fitting a logistic regression model with the linear predictor of the original model and the new marker as predictors:

$$ {\mathrm{lp}}_4=\widehat{\alpha}+{\widehat{\beta}}_{\mathrm{overall}}{\mathrm{lp}}_0+{\widehat{\beta}}_{p+1}m. $$

### Conditional likelihood ratio approach

The conditional likelihood ratio (CLR) approach assumes that the new marker data set contains the same predictors *x*_1_,…, *x*_p_, as the development data set as well as additional information on the new marker *m*. The LR of observing the marker values conditional on the predictors is estimated as:

$$ \mathrm{L}\mathrm{R} = \frac{f\left(m\Big|{x}_1,\dots, {x}_p,\kern0.5em \mathrm{cancer}\right)}{f\left(m\Big|{x}_1,\dots .,{x}_p,\ \mathrm{no}\ \mathrm{cancer}\right)}. $$

The prior odds of having cancer is given by the existing prediction model: Prior Odds = exp (lp_0_). The posterior odds is obtained by combination with the LR using Bayes rule:

$$ \mathrm{Posterior}\ \mathrm{Odds} = \mathrm{Prior}\ \mathrm{Odds} \times \mathrm{L}\mathrm{R}. $$

It has previously been proposed to estimate the components of the LR using linear regression when the marker is measured on a continuous scale [9]. The marker set is split into two separate sets, one containing all patients with cancer versus the other without cancer. A linear regression model is fitted in each set, with the marker as outcome and predictors as covariates. The numerator and denominator of the LR can then be estimated by

$$ \mathrm{L}\mathrm{R}=\frac{\phi_{\mu_{\mathrm{cancer}},{\sigma}_{\mathrm{cancer}}(m)}}{\phi_{\mu_{\mathrm{no}\ \mathrm{cancer}},{\sigma}_{\mathrm{no}\ \mathrm{cancer}}(m)}}, $$

where ϕ is the normal density function, μ_cancer_ and μ_no cancer_ the fitted means of the new marker, and $\sigma$_cancer_ and $\sigma$_no cancer_ the estimated standard deviations of the residuals of the fitted linear regression models for patients with and without cancer, respectively. We label this approach “CLR”.

This approach requires the estimation of 2 (*p* + 1) + 2 parameters, which may result in overfitted prediction models. To limit the number of parameters that need to be estimated the LR may also be estimated using one linear regression model, with the marker as outcome and the predictors of the existing model and an indicator cancer yes/no as covariates (“CLR simple”). The number of parameters estimated using this approach is *p* + 3.

### Imputation approach

In the imputation approach the development and marker set are both used to fit a prediction model containing the predictors of the existing prediction model plus the new marker. This is complicated by the fact that marker values are systematically missing in the development set. We used multiple imputation with chained equations (*mice*) to impute the missing marker value 10 times [16]. In each of the completed datasets a model was fitted with logistic regression. Let ${\widehat{\beta}}_{i,j}$ denote the regression coefficient of predictor *x*_*i*_ in the *j*th completed dataset. The overall estimate of the regression coefficient can be obtained using Rubin’s rules [17]. This overall estimate is simply the average of the estimates in each of the 10 completed datasets:

$$ {\widehat{\beta}}_i=\frac{1}{10}{\displaystyle \sum_{j=1}^{10}}{\widehat{\beta}}_{i,j}. $$

### Model performance

We assessed discrimination and calibration of the extended models. Discrimination refers to the ability of a prediction model to discriminate between patients with and without the outcome of interest. Discrimination was quantified using the concordance statistic (*c*). For a sensible model the *c*-statistic lies between 0.5 and 1. Where 0.5 means that the model does not discriminate better than flipping a coin and 1 means that the model discriminates perfectly. For a logistic regression model the *c*-statistic is equivalent to the area under the ROC curve [18]. Calibration measures the agreement between the predicted probabilities and observed outcomes. Calibration of the extended prediction models was quantified using the calibration slope. Ideally the calibration slope should be equal to 1.0 [5, 7].

### Motivating example continued

The *c* statistic of the ERSPC RC3 was around 0.69 in all five marker sets (Table 3). Extending ERSPC RC3 with PHI typically led to an average increase in c of 0.05, from 0.69 to 0.74, across the different validation sets. In one instance “model revision with extension” and “model extension with shrinkage” did not lead to better discrimination.

**Table 3:** Average and range of c statistic and calibration slopes in the prostate cancer case-study

Measure	Original Model	Model Revision	Model Revision with Shrinkage	Recalibration with extension	CLR	CLR simple	Imputation
*c*-statistic	0.69 [0.68–0.69]	0.73 [0.69–0.75]	0.74 [0.73–0.75]	0.74 [0.73–0.75]	0.74 [0.73–0.75]	0.74 [0.73–0.75]	0.73 [0.73–0.74]
Calibration Slope	0.77 [0.71–0.82]	0.76 [0.42–1.08]	0.75 [0.41–1.06]	0.96 [0.55–1.72]	0.61 [0.54–0.72]	0.74 [0.59–0.91]	0.78 [0.66–0.93]

A previously developed prediction model (RC3) was extended with a marker (PHI) using data from one cohort and validated in four cohorts not used at model development

The calibration of the ERSPC RC3 model with or without the PHI marker was suboptimal in all five marker sets. The calibration slope was smaller than 1, indicating that overall predictor effects were too extreme. Models extended using “CLR” had the poorest calibration slopes, whilst the “CLR simple” and the imputation approach showed slightly better calibration. Calibration slopes of “recalibration with extension” were typically close to one.

### Simulation study

The five datasets containing information on the marker PHI formed the basis for generating simulated development and marker samples. Two settings were simulated, one with the logistic regression model as the true underlying model and one with the “CLR method” as true underlying model. In this way, we allowed for a fair comparison between the two approaches: re-estimation of the regression coefficients and the conditional likelihood ratio approach.

The logistic regression model fitted in the five stacked marker sets was considered the true underlying model (Additional file 1: Table S1). It contained the predictors PSA, prostate volume, DRE and PHI. Patients were drawn with replacement from the five stacked PHI datasets and for each patient the probability of a positive sextant biopsy was calculated with the logistic regression model. The binary outcome variable was generated by comparing the probability of a positive sextant biopsy with an independently generated variable *u*_*i*_ having a uniform distribution from 0 to 1 with Y_i_ = 1 if p_i_ ≥ u_i_ and 0 otherwise.

The true underlying prior model, i.e. without the marker, for the setting with the CLR method was the model fitted in the ERSPC RC3 data. The true underlying linear regression models, that are the components of the likelihood ratio, were the models fitted in the five stacked marker sets, separately for patients with and without cancer (Additional file 1: Table S1).

The binary outcome was generated based on the probability of a positive sextant biopsy given by the prior model, in a similar way as generating the outcome from a logistic regression model. Subsequently, if the generated outcome was a positive biopsy, a value for PHI was generated by drawing a random number from a normal distribution with mean equal to linear predictor of the regression model of PHI for men with a positive biopsy and standard deviation equal to the associated standard deviation of the residuals in the regression model. If the generated outcome was a negative biopsy, the value for PHI was based on the regression model for PHI for men with a negative biopsy.

We considered three scenarios with varying sample sizes of the development and marker samples: 1) 500 for the development sample and 100 for the marker sample, 2) 100 for the development sample and 500 for the marker sample and 3) 500 for the development sample and 500 for the marker sample. In each scenario we generated 1,000 development and marker samples with the same underlying models.

A prediction model containing PSA, prostate volume and DRE was fitted on the development sample and extended with PHI using the marker sample with re-estimation of the regression coefficients or with the conditional likelihood ratio approach. An independent validation sample was generated with 100,000 patients on whom the performance of the extended prediction models was assessed. The validation sample was generated using the same models as the development and marker sample. Performance measures considered were the *c*-statistic and the calibration slope. All simulations were done using R 2.14.1 [19], with multiple imputation (10 times) using the *mice* package [20]. R-scripts used in the simulation studies are available online (Additional files 2 and 3).

## Results 

**Fig. 2:**
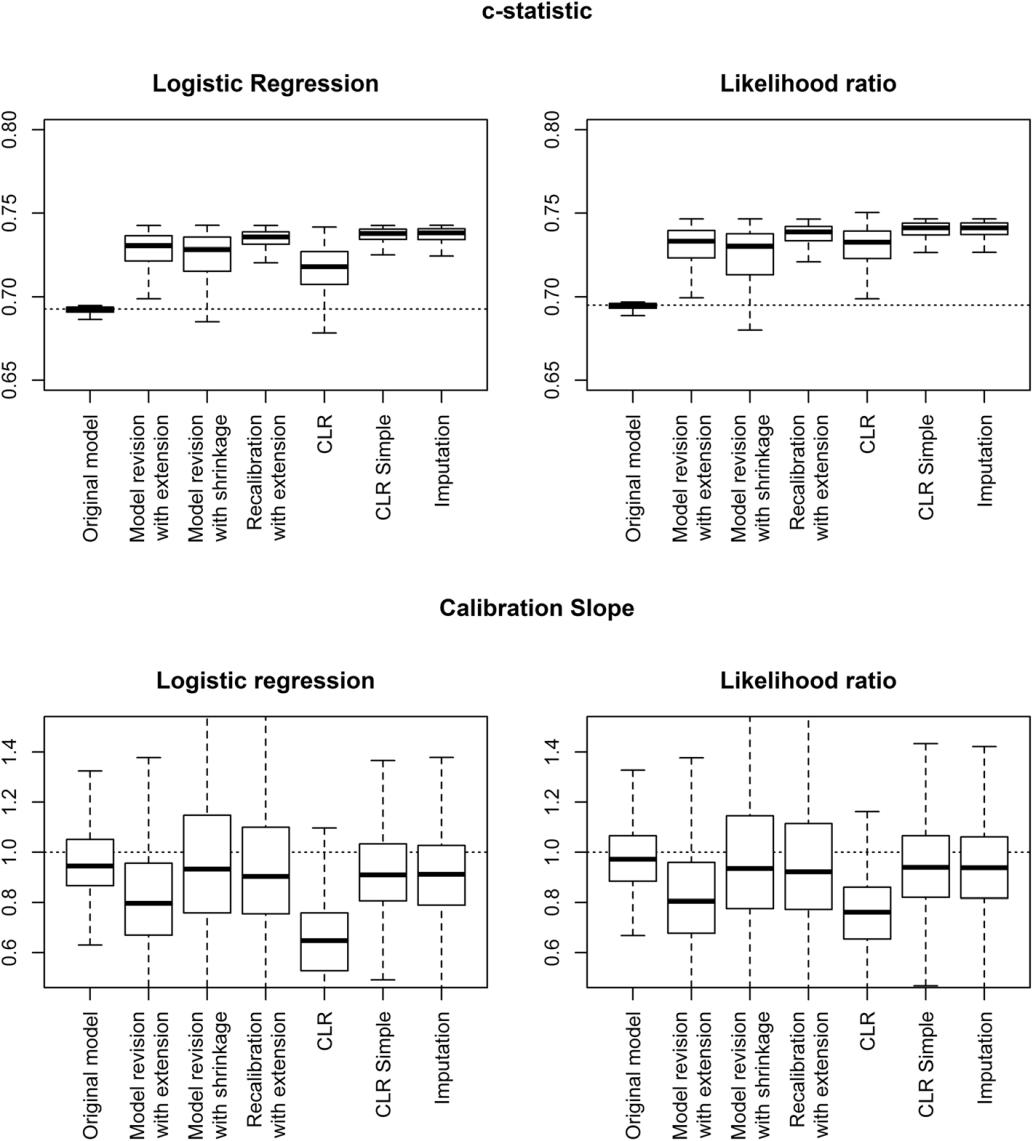
Calibration slope and c-statistic of updated models in a simulation study for development set size 500 and marker set size 100. Outcomes were generated using a logistic regression model and the CLR model as the underlying true models. Box plots are based on 500 simulations

When the development sample contained 500 patients and the marker sample 100 patients, the models without the marker PHI showed a *c*-statistic of 0.69 in the development samples (Fig. 2). Extending the prediction model with the marker showed a *c*-statistic of around 0.73. “Recalibration with extension”, “CLR simple”, and the “imputation approach” led to the largest increase in *c* with relatively low variation.. Models extended with “model revision with extension” and “CLR” showed calibration slopes below one, indicating overfitting in the small marker samples. Other extension methods showed median calibration slopes closer to one, similar to the model without the marker. The choice of true underlying model only influenced the model performance for the “CLR” method, both in discriminative ability and calibration and in all scenarios.

**Fig. 3:**
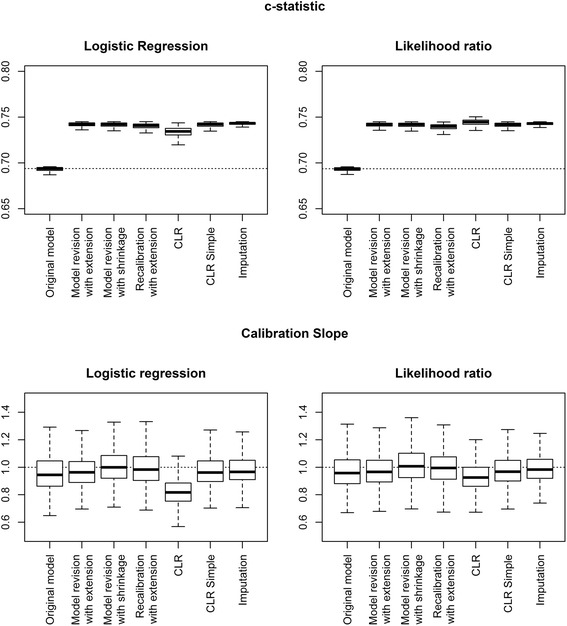
Calibration slope and *c*-statistic of updated models in a simulation study for development set size 500 and marker set size 500. Outcomes were generated using a logistic regression model and the CLR model as the underlying true models. Box plots are based on 500 simulations

When the marker sample was larger, 500 patients, with the same development sample size of 500, the variation in *c*-statistic was much lower compared to the scenario with a development sample of 500 and a marker sample of 100 (Fig. 3).

The median calibration slopes were closest to one for the methods “model extension with shrinkage” and “recalibration with extension”. The median calibration slope of the models extended using “CLR” was well below one.

**Fig. 4:**
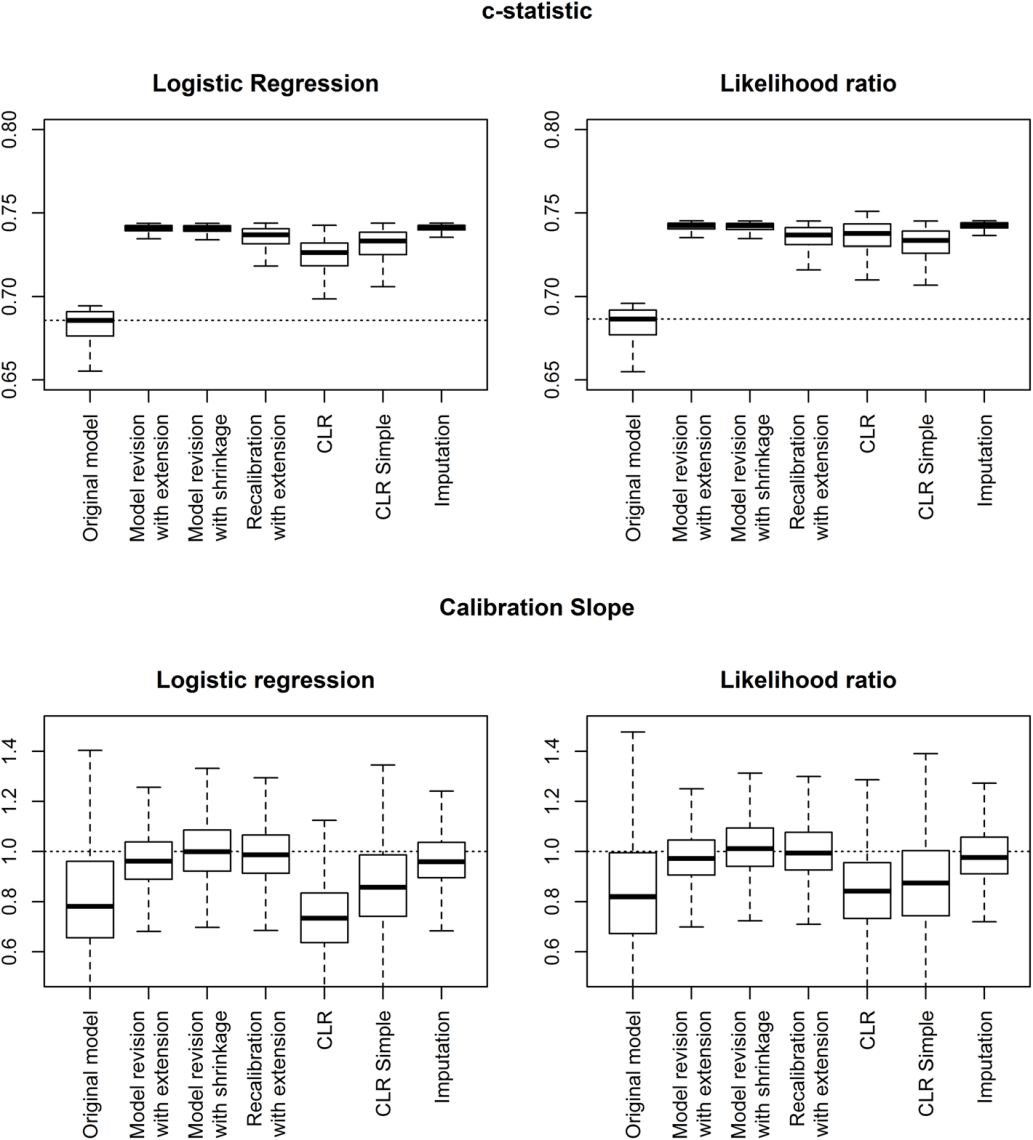
Calibration slope and *c*-statistic of updated models in a simulation study for development set size 100 and marker set size 500. Outcomes were generated using a logistic regression model and the CLR model as the underlying true models. Box plots are based on 500 simulations

When the development sample contained only 100 patients and the marker sample 500 patients, more variation in *c*-statistic was found than in the other scenarios reflecting the smaller sample size at development (Fig. 4). The methods “model revision with extension”, “imputation”, and “model revision with shrinkage” showed the largest values for the *c*-statistic.

The median calibration slope of the prediction models without the marker PHI was well below one, reflecting too extreme predictions due to the small development samples. Re-estimation of the regression coefficients and the “imputation” approach improved the calibration slopes with values close to one. “CLR” and “CLR simple” showed median calibration slopes well below one.

## Discussion

We compared different strategies of extending an existing prediction model with a new marker. We found that when the dataset used to extend the prediction model was small, parsimonious methods led to the largest increase in discriminative ability of the prediction model, but as the available sample size to extend the prediction model increased more extensive extension methods outperformed parsimonious methods. Strategies requiring the estimation of many parameters, such as Bayesian updating with conditional likelihood ratios estimated per outcome (“CLR”) and “model revision with extension”, resulted in too extreme predictions. Strategies that required estimation of fewer parameters, such as “recalibration with extension” or “CLR simple”, and strategies that applied shrinkage, all resulted in well-calibrated predictions for new subjects. The “imputation strategy” also required the estimation of a relatively large number of parameters, i.e. all individual regression coefficients, but the combination of the development and marker sets led to the largest data set possible. This strategy led to more precise estimates of regression coefficients, and consistently well performing prediction models.

The structure for simulating the datasets was based on empirical data, which simplified the data generation procedure and avoided arbitrary choices in predictor distributions and predictor effects [21]. The outcomes were generated from a logistic regression model or from a model that was consistent with the CLR method. This allowed for a fair comparison between prediction models based on logistic regression and based on the CLR methods. As expected, the CLR methods showed lower performance when the true underlying model was a logistic regression model. The methods that fitted logistic regression models were less sensitive to the underlying model generating the outcome.

Our simulation study used patients from one homogeneous underlying population, meaning that the predictor effects in the development and marker sets were assumed to be similar. In practice this may not be the case. Predictor effects in the development and marker sets may be truly different (heterogeneity), or the prediction model may have been overfitted at development [22]. Both heterogeneity and overfitting lead to prediction models with incorrect regression coefficients when applied in the marker set. Methods using conditional likelihood ratios to update predictions do not adjust predictor effects of the existing model. These methods are hence not useful to extend models that have incorrect regression coefficients for the marker set.

Our case study was based on a widely used risk prediction tool for prostate cancer. We illustrate that adding a new marker to such an existing prediction model may lead to substantially better model performance, in particular better discrimination. We recognize that multiple markers may be available, all with the potential to improve discrimination. For parsimony, markers can be selected in a stepwise forward manner [8]. Or multiple markers can be combined in a simple summary score, with the summary score added as a single predictor. This approach was followed for the PHI marker which consists of a combination of the biomarkers PSA, free-PSA and [−2] proPSA [13].

We compared the performance of prediction models in terms of calibration and discrimination. Recently, other measures for clinical usefulness have been suggested to assess the added value of markers, e.g. the net reclassification index (NRI), net benefit, and relative utility [23–25]. All these measures consider the number of true positives and true negatives at particular risk thresholds and are sensitive to the calibration of a prediction model. Miscalibrated prediction models might even show misleading performance when calculating the NRI [26, 27]. The risk of overfitting should hence not be taken lightly as induced by simply refitting a model in a small data set where a new marker is available.

When possible, combining the development and validation sets is preferable, since this uses the full information available in the development set and consequently limits the risk overfitting.

A limitation of this study is that we considered a case study in which relatively few regression coefficients were estimated in the original model (3 in total). We expect that differences between the different strategies would become clearer when considering prediction models containing larger number of predictors, or when smaller marker sets are considered.

## Conclusion

This study shows that the “imputation approach” is a suitable strategy to improve prediction models with new markers. This approach combines the data set used at development of the existing prediction model with the new marker data set. With access to only a small marker data set, we recommend parsimonious methods, such as “recalibration with extension” and “CLR simple”. Larger marker data sets allow for more extensive updating of the prediction model using “model revision with shrinkage”.

## Additional files

Additional file 1: Table S1. Coefficients used for generating datasets in the simulation study. Table containing the coefficients used in generating datasets in the simulation study. (DOCX 13 kb) Additional file 2: Script 1. R-scripts for running the simulation study. (R 9 kb) Additional file 3: Script 2. R-script containing functions used for running the simulation study. (R 2 kb)

## Abbreviations

CLR: Conditional likelihood ratio
DRE: Digital rectal exam
ERSCP: European randomized study of screening for prostate cancer
LR: Likelihood ratio
PHI: Prostate health index
PSA: Prostate-specific antigen
RC: Risk calculator
