# Supplementary material for: Improving prediction models with new markers: a comparison of updating strategies
Source: BMC Med Res Methodol. 2016 Sep 27;16:128. doi: 10.1186/s12874-016-0231-2 (PMC5039804; doi:10.1186/s12874-016-0231-2)
Supplement: Additional file 1: Table S1. — Coefficients used for generating datasets in the simulation study. Table containing the coefficients used in generating datasets in the simulation study. (DOCX 13 kb) [file 12874_2016_231_MOESM1_ESM.docx]

|  | Coefficients of logistic regression model |  | Coefficients of models for CLR method |  |  |  |
| --- | --- | --- | --- | --- | --- | --- |
|  |  |  | Prior model |  | PHI model for men with cancer | PHI model for men without cancer |
| Intercept | -5.31 |  | -0.24 |  | 5.53 | 5.25 |
| PSA | 0.19 |  | 0.60 |  | 0.56 | 0.33 |
| Prostate volume | -1.09 |  | -1.41 |  | -0.37 | -0.30 |
| DRE | 0.52 |  | 0.67 |  | 0.19 | 0.12 |
| PHI | 0.94 |  | - |  | - | - |
| SD residuals | - |  | - |  | 0.68 | 0.59 |
